# The Evaluation of Albumin Utilization in a Teaching University Hospital in Iran

**Published:** 2011

**Authors:** Zahra Jahangard-Rafsanjani, Mohammad Reza Javadi, Hassan Torkamandi, Sara Alahyari, Azita Hajhossein Talasaz, Kheirollah Gholami

**Affiliations:** a *Tehran University of Medical Sciences, Tehran, Iran.*; b *Department of Clinical Pharmacy, School of Pharmacy, Tehran University of Medical Sciences, Tehran, Iran. *

**Keywords:** Utilization evaluation, Albumin, Teaching hospital

## Abstract

The goal of this study was to evaluate the pattern and the suitability of the human Albumin usage according to the available and reliable guidelines.

A concurrent, cross-sectional study was performed in Shariati Hospital (associated with Tehran University of Medical Sciences, Tehran, Iran). All inpatient adults that were prescribed albumin during the study period were evaluated to register the indications for albumin usage and specific patient information. The total 1281 vials of Albumin were prescribed for 135 patients during the study period. The most common reasons to prescribe albumin were volume expansion after the heart surgery (53.3%), nutrition source in malnourished patients (19.3%), paracentesis (12.9%), plasmapheresis (9.6%), hypoalbuminemia (3%) and the others (2.1%). Only 411 vials (32.1%) prescribed with 34 prescriptions (25.2%) were utilized appropriately based on the guidelines.

The results showed that based on the guidelines, the most prescriptions of albumin in this hospital have not been written appropriately. Therefore, educational programs on using guidelines may help reduce albumin usage and treatment costs.

## Introduction

Irrational drug usage is one of the most important problems in the health system. The rational use of medications is based on the appropriate guidelines and clinical needs of patients. Furthermore, medications should be prescribed in doses that meet patient’s individual requirements, for an adequate period of time at the lowest cost for them and their community (1). Human albumin is an expensive medication due to the limited supply and difficulty in production. Albumin Drug Utilization Review (DUR) has reported this product is not used appropriately; in some studies, more than 90% of albumin prescriptions are inappropriate (2). It is therapeutically, economically and academically essential to ensure that this drug is administered appropriately. According to the reliable guidelines, there are some inexpensive alternatives as first-line therapy in many clinical conditions. Moreover, there are clinical conditions and/or procedures such as plasmapheresis and paracentesis that require albumin as the first line therapy.

According to the statistics put out by the Food and Drug Organization of Health Ministry on the Human albumin, 472089 vials albumin 20% costing $ 21,600,000 have been used in the first 9 months of 2008. This is the highest cost for a single drug used in the hospitals of our country. At the same time in our hospital (Shariati hospital) which is a teaching hospitalof Tehran University of Medical Sciences, about 10800 vials of albumin 20% were used at the cost of $ 496800. This has been the highest cost for a medication used in our hospital. Based on this information, we decided to perform a DUR on albumin to determine whether the usage of albumin is rational (according to the guidelines).

## Experimental

This study was a concurrent and cross-sectional DUR that performed from 30^th^ May to 30^th^ June 2009 in inpatient wards of Shariatee hospital. This hospital is a general teaching university hospital with 38 wards and 477 beds with the most medical specialties. A standard form on albumin indications was designed based on reliable guidelines (3-7). Daily list of adult patients with albumin prescription was obtained from inpatient pharmacy. Two pharmacist from pharmacovigilance service and drug information center using the form, gathered data on reasons for the prescription, the amount used, the administration length, the potential contraindications and the possibility of using the other alternative treatments. In this study, a second form was filled for the patients who were prescribed albumin for two different indications. At the end of the study period, the collected data were analyzed. The prescriptions which were matched with the guidelines were considered appropriate. The summary of guidelines used in this study is shown in Table 1. 

**Table 1:** Summary of implied guidelines in this study

**Indication **	**Notes**
Ascitic patient post paracentesis (2, 7)	Five g of albumin/L ascitic fluid was removed after the paracentesis of volume > 4 L.
Therapeutic plasmaphresis (3-7)	Albumin is appropriate for the exchanges in the range of > 20 mL/Kg in one session or the range of > 20 mL/Kg/week in more than one session.
Spontaneous bacterial peritonitis (7)	Albumin is appropriate in association with antibiotic.
Cardiac surgery (3, 4, 5, 7)	Crystalloids are the first choice as the priming solution for cardiopulmonary bypass pumps. The use of non-protein colloids in addition to crystalloids may be preferable in cases in which it is extremely important to avoid pulmonary edema. For postoperative volume expansion, crystalloids should be considered first-line therapy, followed by non-protein colloids, and albumin is the last choice.
Hemorrhagic Shock (3-7)	Crystalloids should be considered the first choice for resuscitation. When crystalloids (4 L) have failed to produce a response within 2 h for adult patients, non-protein colloids may be considered. When non-protein colloids are contraindicated, albumin 5% may be used.
Non-hemorrhagic shock (3-6)	Albumin is the last choice after crystalloids and non-protein colloids.
Nephritic Syndrome (3-7)	Diuretic therapy is the initial treatment. The short-term use of albumin 25% with diuretic therapy is appropriate for the patients with acute severe peripheral or pulmonary edema who have failed diuretic therapy.
Severe burn (5-7)	Fluid resuscitation should be initiated with crystalloid solutions. If crystalloid resuscitation exceeds 4 L in adults 18 to 26 h post-burn and burns cover more than 30% of the patient’s body surface area, non-protein colloids may be added. If non-protein colloids are contraindicated, albumin may be used.
Liver transplantation (3, 5, 7)	Albumin may be used in post-operative period to control ascites and peripheral edema if the following conditions are met: 1. Serum albumin less than 2.5 g/dL; 2. Pulmonary capillary wedge pressure less than 12 mmHg; 3. Hematocrit greater than 30%.
Albumin indications considered inappropriate (3-7)	Hypo-albuminemia Nutritional Interventions Impending hepatorenal syndrome Increasing drug efficacy Acute/chronic pancreatitis

## Results and Discussion

During the study 135 patients received albumin vials. Seventy-six patients (56.3%) were male and 59 (43.7%) were female. Patients’ mean age ± SD was 48.7 ± 15.6 years (in the age range of 18-82 years). The total number of albumin vials used was 1281 with a mean of 9.4 (SD = 14.03; Median = 4; Quartile 1 = 2; Quartile 3 = 12) vials per patient, ranging from 1 to 90 vials. During this study, the patients in 11 wards out of 38 wards of this hospital received albumin, among them, most prescriptions were from ICU-open heart (54.8%) and the respiratory ward had the least prescriptions (0.7%). The distribution of albumin prescriptions in different wards is shown in Table 2. 

**Table 2:** Distribution of albumin prescriptions in different wards

**Number of prescriptions (%)**	**Number of vials (%)**	**Ward**
17 (12.6)	328 (25.6)	Surgical recovery
74 (54.8)	283 (22.1)	ICU-open heart
8 (5.9)	209 (16.3)	ICU-general
6 (4.4)	98 (7.6)	Neurology
2 (1.5)	87 (6.8)	ICU-neurosurgery
1(0.7)	71 (5.5)	Respiratory ward
5 (3.7)	56 (4.4)	Gastroenterology and endocrinology
8 (5.9)	52 (4.0)	Women and delivery ward
3 (2.2)	45 (3.5)	Oncology
6 (4.4)	31 (2.4)	Emergency
2 (1.5)	21 (1.6)	Nephrology
135 (100)	1281 (100)	Total

The reasons for the albumin prescribed were as follows: volume expansion after cardiac surgery 72 (53.3%), nutrition source in malnourished patients 26 (19.3%), paracentesis 17 (12.6%), Plasmapheresis 13 (9.6%). Other indications such as hypoalbuminemia, nephrotic syndrome and hemorrhagic/non-hemorrhagic shock all together comprised 7 (5.1%) of the total. The number of vials used for each indication was not proportional to the number of prescriptions. The collected data reports 594 vials (46.4%) in malnourished patients for nutritional supply, 235 vials (18.3%) in Plasmapheresis, 215 vials (16.8%) for volume expansion after cardiac surgery, 146 vials (11.4%) for paracentesis and 91 vials (7.1%) for other indications.

We also reported that-based on the guidelines in the study–only 34 prescriptions (25.2%) with sum off 411 albumin vials (32.1%) were administered appropriately and the rest were inappropriate. The appropriate use of albumin occurred most frequently in paracentesis (15 cases) and Plasmapheresis (13 cases). Albumin administered for volume expansion after the cardiac surgery in 69 cases was most inappropriate. The details of the above findings are shown in Table 3.

**Table 3:** Characteristics of albumin usage by reasons of indication

**Indication**	**Appropriate use**		**Inappropriate use**	
	**Number of patients (%)**	**Number of vials (%)**	**Number of patients (%)**	**Number of vials (%)**
Cardiac surgery	3 (2.2)	16 (1.2)	69 (51.1)	199 (15.5)
Paracentesis	15 (11.1)	137 (10.7)	2 (1.5)	9 (0.7)
Plasmapheresis	13 (9.6)	235 (18.3)	-	-
Hemorrhagic shock	1 (0.7)	3 (0.2)	-	-
Non-hemorrhagic shock	1 (0.7)	9 (0.7)	-	-
Nutrition	-	-	26 (19.3)	594 (46.4)
Hypoalbuminemia	-	-	4 (3)	68 (5.3)
Nephrotic syndrome	1 (0.7)	11 (0.9)	-	-
Total	34 (25.2)	411 (32.1)	101 (74.8)	870 (67.9)

Results from this study show that more than three-fourths of albumin prescriptions corresponding to 870 (67.9%) vials of albumin have not been administered appropriately based on the reliable guidelines used in this hospital. Similar results have been obtained in other comparable studies; Tanzi *et al.* (in 2000) evaluated albumin usage in 53 institutes in United States and found that 57.8% of albumin prescriptions in adult patients were inappropriate (8). Furthermore, the result of a study in Spain indicated that more than 90% of albumin administrations were not according to Vermeulen *et al.* recommendations, the university hospital consortium guidelines for the use of albumin,

non-protein colloids (NPCs) and crystalloid solutions (2).

In our study, the albumin indication after the cardiac surgery has been the most inappropriate prescriptions among other indications. According to the majority of reliable guidelines, albumin is recommended as the last choice, after crystalloid solutions and NPCs, after the open-heart surgery volume expansion. In our hospital, NPCs have not been used at all and albumin is used as the second line of therapy after the crystalloid solutions. There is a controversy about albumin and NPCs usage for volume expansion after Coronary Artery Bypass Graft Surgery. The research of Sedrakyan *et al.* published in 2003 showed that the albumin usage as volume expanding after Coronary Artery Bypass Graft Surgery instead of NPCs can reduce mortality rate by 25% after surgery (9).

A systematic review of 14 studies about albumin usage as volume expander published in 2003, compared albumin with crystalloids and NPCs. The result of this review showed that the administration of albumin reduced the necessity of fluid therapy in comparison with crystalloids and NPCs. The adverse effects observed by crystalloids, such as respiratory impairment and pulmonary edema, have not been reported during the albumin usage. These adverse effects may be attributable to the crystalloid-mediated reductions in colloidal oncotic pressure (COP) and COP-pulmonary arterial wedge pressure (PAWP) gradient. Nevertheless, there is no solid study to confirm the preference of colloids to crystalloids in cardiac surgery (10, 11). Therefore, there is no consistency in the usage of albumin, crystalloids and NPCs for volume expansion after the Coronary Artery Bypass Graft Surgery and cardiac surgery sites have different views in this regard. For example, in the survey by Kustrup *et al.* three-fourths of ICUs in Germany indicated the colloidal solutions as the first-line therapy and 90% of them administered NPCs as colloidal solution after the cardiac surgery. Only 21% use the crystalloids for volume replacement as the first-line treatment (12).

On the other hand, according to the guidelines used in this study, using albumin immediately after the crystalloid solutions was considered inappropriate in most cases, with the exception of 3 patients who had contraindication to NPCs.

Some studies have shown that the low serum albumin level is associated with poor outcome in critically ill patients and perhaps, this is the reason why physicians tend to prescribe albumin in hypoalbuminemia. But in several studies, it has been shown that the albumin administration in a patient with hypoalbuminemia did not have distinctive effect on mortality and morbidity (13). Therefore, in most guidelines hypoalbuminemia is an inappropriate indication for the administration of albumin and just in some cases that the serum albumin level is less than 2.5 g/dL, albumin can be prescribed (7, 14).

In this study, none of the patients had serum albumin levels less than 2.5 g/dL and the indication of albumin for hypoalbuminemia was considered inappropriate for them. In a similar albumin utilization study at a private hospital in Bangkok, 32 patients received albumin for hypoalbuminemia among which 11 had serum albumin level more than 3 g/dL, hence, 35% of albumin administration in hypoalbuminemia was inappropriate (14).

According to the guidelines, the albumin administration in hemorrhagic shock is rational when crystalloids have failed to produce a response and NPCs are contraindicated. In the present study, just one patient received albumin for hemorrhagic shock. The patient had renal insufficiency, so, NPCs could not be used and albumin usage was appropriate in this case. In a comparable study in Spain, 50% of cases in hemorrhagic shock crystalloids and colloids were administered and in spite of good response to these products, albumin was administered as well (2).

In paracentesis, albumin administration is within the recommended guidelines when a large volume of ascetic fluid has been removed. This could be from 2 to 5 L in various guidelines. In this study, the administration of albumin was considered appropriate to remove 4 L of ascetic fluid. In two cases, this was less than 4 L, so, albumin usage in these patients was considered inappropriate and in other cases was considered appropriate. The administration of albumin in Plasmapheresis in all cases was appropriate in our study.

One of the irrational usages of albumin is to prescribe this product in malnourished patients for nutritional support. In the current study, 46.6% of albumin administer for this inappropriate indication. For malnourished patients, the correct treatment is enteral and/or parenteral nutrition. However, albumin may be used in patients with diarrhea associated with intolerance to enteral feeding if all of the following conditions are met: 1) diarrhea volume more than 2 L/day; 2) serum albumin less than 2 g/dL; 3) continuing diarrhea despite administration of short-chain peptides and elemental formula (15). None of the patients in our study had these conditions for albumin usage in nutritional support. Similar results are found in other studies; for example, in two university hospitals in Spain, 100% of albumin usage in malnourished patients was inappropriate (2).

In conclusion, based on reliable guidelines, a large number of albumin administrations in Shariatee hospital are not rational. Even ignoring controversial indications such as volume expansion after cardiac surgery, about 50% of albumin is administered inappropriately for hypoalbuminemia and nutritional support. Therefore, an educational program on introducing and implementing appropriate guidelines may help to reduce albumin usage and treatment cost in this center. However, the implementation of this program is complex because of some inconsistency in the literature on the real indications of this product.

## Conclusion

This type of review can alert the physicians to change the pattern of prescriptions, especially for more expensive and frequently used medications. As a result, it is necessary to perform another DUE after the educational program on introducing and implementing appropriate guidelines to evaluate the impact of these interventions on the reduction of albumin usage.
